# Nanotechnology: new opportunities for the development of patch‐clamps

**DOI:** 10.1186/s12951-021-00841-4

**Published:** 2021-04-01

**Authors:** Jia Gao, Chunyang Liao, Sijin Liu, Tian Xia, Guibin Jiang

**Affiliations:** 1 State Key Laboratory of Environmental Chemistry and Ecotoxicology, Research Center for Eco-Environmental Sciences, Chinese Academy of Sciences, Beijing, 100085 China; 2 College of Resources and Environment, University of Chinese Academy of Sciences, Beijing, 100049 China; 3 Division of NanoMedicine, Department of Medicine, University of California, Los Angeles, CA 90095 USA

**Keywords:** Electrophysiology, Intracellular recording, Nanoelectrode, Field‐effect transistors (FETs), Neuronal activity

## Abstract

The patch-clamp technique is one of the best approaches to investigate neural excitability. Impressive improvements towards the automation of the patch-clamp technique have been made, but obvious limitations and hurdles still exist, such as parallelization, volume displacement in vivo, and long-term recording. Nanotechnologies have provided opportunities to overcome these hurdles by applying electrical devices on the nanoscale. Electrodes based on nanowires, nanotubes, and nanoscale field-effect transistors (FETs) are confirmed to be robust and less invasive tools for intracellular electrophysiological recording. Research on the interface between the nanoelectrode and cell membrane aims to reduce the seal conductance and further improve the recording quality. Many novel recording approaches advance the parallelization, and precision with reduced invasiveness, thus improving the overall intracellular recording system. The combination of nanotechnology and the present intracellular recording framework is a revolutionary and promising orientation, potentially becoming the next generation electrophysiological recording technique and replacing the conventional patch-clamp technique. Here, this paper reviews the recent advances in intracellular electrophysiological recording techniques using nanotechnology, focusing on the design of noninvasive and greatly parallelized recording systems based on nanoelectronics.
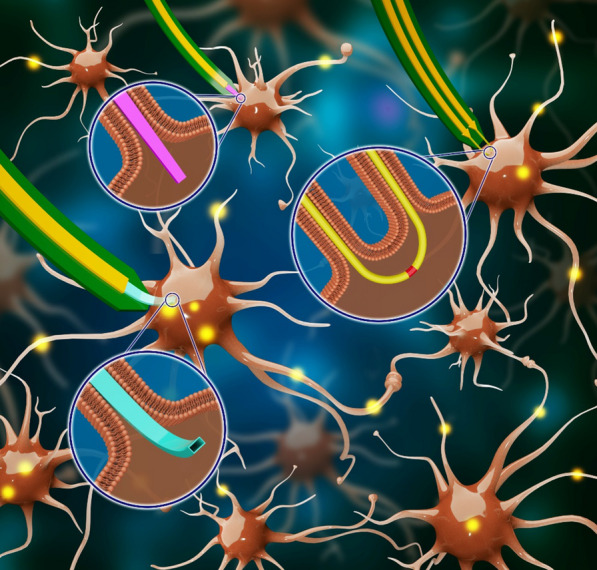

## Introduction

Direct measurements of the cell membrane potential play a fundamental role in studying neurons and other electrically excitable cells and thus are a central concern of neurophysiology and cardiology [1, 2]. In terms of neurons and cardiomyocytes, the spontaneous action potential provides abundant information about the type, state, and properties of ion channels located on the cell membrane, the dysfunction of which is associated with several neural and cardiac diseases [3, 4]. Patch-clamp technique establishes direct contact with the intracellular environment through the penetration of a glass micropipette, and the formation of a high-resistance seal minimizes the perturbation of the extracellular potential, allowing for high-precision recording of the intracellular potential [5, 6]. To date, the patch-clamp technique is the most sensitive approach among all intracellular recording methods, providing information about neural electrical activity with a high temporal resolution and high signal-to-noise ratio. However, the conventional patch-clamp technique is difficult to implement, highly invasive, and non-compatible for high throughput screening and multiplexing [7]. In the case of the traditional manual patch-clamp technique, several steps in forming a stable seal are highly time-consuming and difficult, which limits the number of data points obtained each day [8]. Recently, the development of the planar mode of the patch-clamp technique has achieved substantial advances in increasing the throughput, and the automation of the major steps in the patch-clamp technique reduces the manipulation difficulty [9]. Despite this considerable progress, the automated patch-clamp system has high invasiveness during recordings and obvious limitations in cell preparation. Currently, the highly invasive penetration of micropipettes has not changed in these systems and will lead to dialysis and interdiffusion between the cytoplasm and pipette solution, thus disrupting the natural electrophysiology of the cell during measurement. Another obvious limitation of the patch-clamp technique is the potential of multiplexing, which is vital for studies about neuronal networks [10]. Several factors of the patch-clamp system account for the present challenges in its parallelization, including the size of the micropipette, the rigid structure, and the necessary microfluidic system behind the pipette [11]. To be more concrete, the size of the glass micropipettes widely used in most of traditional patch-clamp machines is about several microns. However, due to their rigid structure, the patch-clamp micropipette, the linked electronics and the microfluidic system (supporting the continuous patch-clamp recordings) occupy a large volume and prevent the application in studies on neuronal networks, which need spontaneous measurement of multiple neurons within a small range [11, 12]. Therefore, recordings targeting specific small cells or subcellular structures are not possible in patch-clamp systems.

The past few decades have witnessed the rapid development of nanotechnology. According to previous practices, the combination with nanotechnology would be a practical orientation for the development of noninvasive and parallelized patch-clamp electrophysiology. Several configurations of nanoelectrodes or nanotransistors have been used to measure the intracellular potential and achieve considerably high recording quality comparable to conventional patch-clamp techniques [1, 13]. Compared to conventional pipettes, nanoelectrodes are less invasive and easily reproducibly constructed and densely mapped for multiplexing. Vertical metal nanowire electrode arrays have achieved true intracellular recordings from several mammalian cell lines. The individual metal electrode of the nanowire array, ranging from 10 nm to ~ 1 μm in diameter, can be produced using nanofabrication technology in a massively parallelized manner on a planar substrate [12]. These subcellular-sized nanowire probes can contact many cellular and subcellular structures without ion exchange with the cytoplasm. The integration of nanowire arrays with complementary metal-oxide-semiconductor (CMOS) circuits enables further increasing the number of electrodes integrated on the same substrate and optimizes the signal collection [14]. The nanoscale field-effect transistor has a small detector region located at the tip of the kinked structure and enables less invasive entry into the cell. Compared to metal nanopillar electrodes, nanotransistors process information from the source and the drain without direct electrical connections with the intracellular environment, thus avoiding increased signal distortion as the electrode diameter decreases in the design of metal nanoelectrodes [15, 16]. In addition, nanotube-based electrodes and extracellular nanoelectrodes simulating intracellular recordings also show considerable advances in reducing invasiveness, increasing the signal-to-noise ratio and multiplexing [17]. In the following sections, we will discuss the recording mechanisms, the electrical properties and the major limitations of these nanoscale devices, focusing on their potential to establish a scalable, high-precision and long-term recording platform with high temporal and spatial resolution.

## Development of electrophysiological recording methods

### Patch‐clamp

**Fig. 1:**
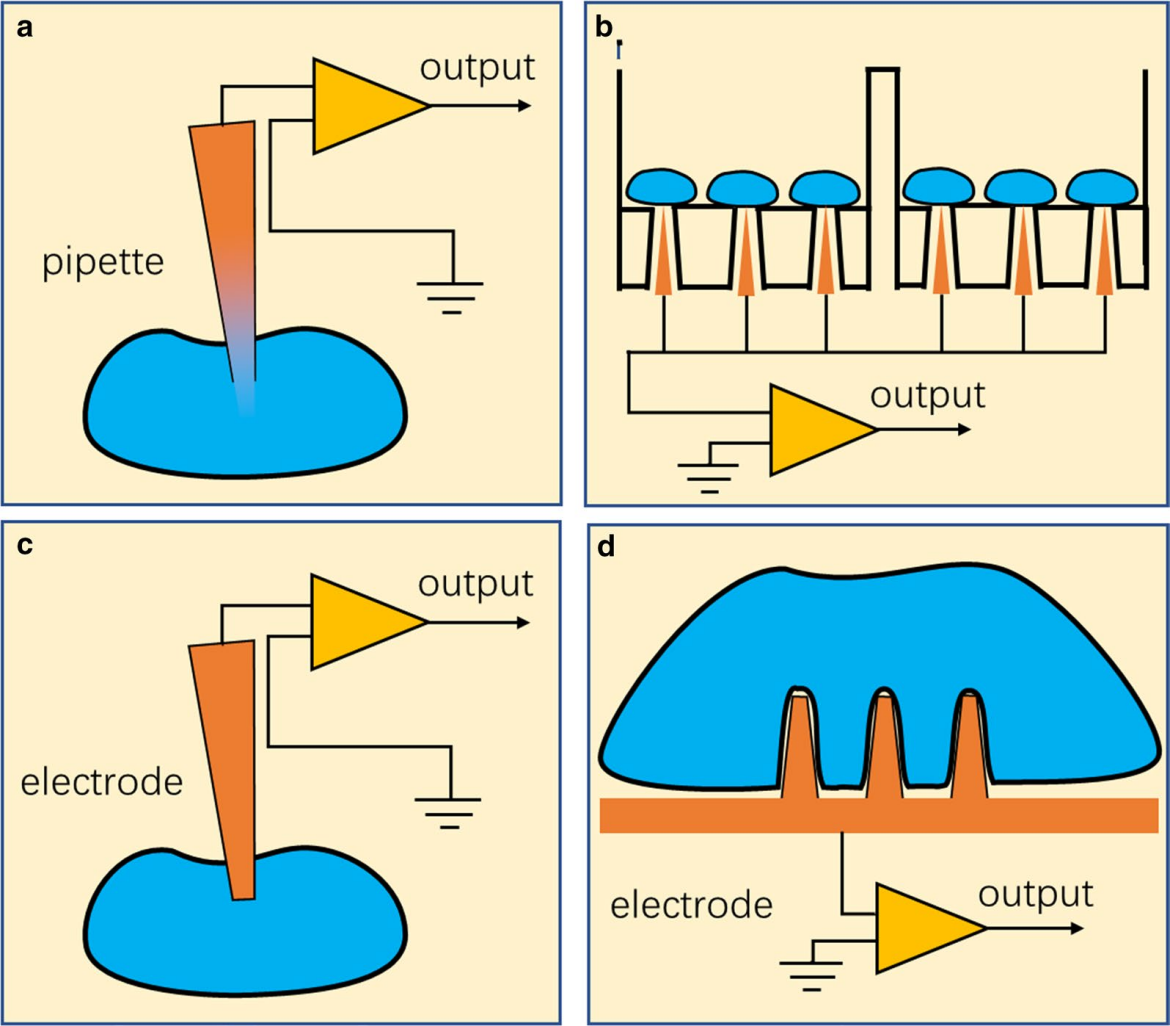
Four forms of intracellular recording platforms. **a** Glass micropipette in patch-clamp, adapted from ref. [7]. **b** Planar patch-clamp system, reproduced from ref. [22]. **c** Sharp electrode, reproduced from ref. [22] with permission. **d** Microelectrode array, adapted from ref. [7]

The origin of intracellular recordings dates back to the 1940s when the voltage-clamp method was utilized to measure the transmembrane current of the neuronal fiber by Hodgkin and Huxley [18]. Using the same electrical principles as a voltage-clamp, a patch-clamp system is capable of clamping the membrane voltage through the impalement/attachment of a glass micropipette with a micron size diameter to the membrane. In terms of the whole-cell patch-clamp configuration, the membrane patch under the tip of micropipette is broken and the electrode inside the micropipette simultaneously achieves an electrical connection with the cytoplasm to measure the intracellular transmembrane potential (Fig. 1a) [19]. Light suction is applied to keep the intense contact between the pipette and the cell membrane, aiming to establish a resistance seal and reduce the noise. The high temporal resolution and high-precision recording statistics of the intracellular transmembrane potential provided by the patch-clamp technique were used as the basis of a large number of previous studies in neuroscience, leading to new observations of ion channel properties and functions. Though intracellular recording with the patch-clamp pipette has been the gold standard in studying cellular electrical activities and ion channel properties, this method has barely changed in recent decades and has always been critiqued for having many obvious disadvantages, such as its manual manipulation, low throughput, volume occupation and high invasiveness [20].

To date, using the conventional patch-clamp platform has been complained for its low efficiency in neurophysiological studies and pharmaceutical screening, which is one of the most important motivations for a novel intracellular recording system [7, 21, 22]. There are several progresses in automating the steps of patch-clamp recordings and these automatic systems truly advanced the performance of patch-clamp technique by improving the availability and stability and reducing the difficulty of the technique. The automated patch-clamp systems in vitro have developed several special configurations that allow for the fast and standard screening of compounds [23]. Automated patch-clamps on planar substrates have combined the conventional recording mode with the microfluidic approach, exceeding the throughput of conventional systems through innovations in cell capturing. Several automated planar patch-clamp systems have achieved various levels of advances in automation and parallelization. Compared to conventional recording systems, automated patch-clamp systems arrange multiple pipettes on a planar chip, and the suspended cells are delivered to recording sites through a microfluidic system and form the resistance seal (Fig. 1b) [24]. Many previous studies have demonstrated that this recording method can provide high-precision whole-cell patch measurement information comparable with that of conventional patch-clamp systems [3, 25, 26]. With increased data throughput and reduced technical requirements, automated planar patch-clamp systems have shown significant potential in research on ion channels related to drug discovery.

### Sharp microelectrode

Sharp microelectrode recording is a traditional intracellular recording method that accesses the cytoplasm through the impalement of solid electrodes [27]. Sharp microelectrodes are directly advanced into the cells of interest through mechanical force to obtain the intracellular signals (Fig. 1c). Recording using sharp electrodes applies smaller diameter electrodes during experiments and prevents diffusion between the pipette solution and cytoplasm compared to the patch-clamp technique. However, this method was rapidly replaced by intracellular recording in vitro and in vivo because this direct recording mode probably resulted in much more damage to the recorded cells and disrupted the physiological properties [15]. In the recordings of neurons, especially the species that have high input impedance, the penetration of sharp electrodes significantly alters the electrical properties of the recorded neurons, including reducing the resting membrane potential and decreasing the neuron input resistance. These changes are attributed to the leakage of the cytoplasm and the shunt current at the penetration site. Moreover, it has been reported that the impalement of the cell membrane was harder for sharp electrodes compared to patch-clamp pipettes, and a series of spikes followed by sustained depolarization were observed after unsuccessful impalements. Due to the disruptive effects, the application of sharp electrodes in intracellular recording has been rapidly replaced by the whole-cell patch-clamp technique [27].

### Microelectrode array

The microelectrode array is a typical extracellular recording method that arranges large amounts of microelectrodes on a planar substrate, moves these electrodes to make their tips approach the cell surface, and records the extracellular local field potentials (Fig. 1d) [28]. This method is a noninvasive recording technique widely used in studies on the cell network. The microelectrode array recording system obtains information about neuronal electrical activity through the measurement of local field potentials. Because spike activities, action potentials, and synaptic potentials simultaneously lead to intracellular and extracellular voltage changes, microelectrode arrays can quickly catch the signals and reconstruct the reasons for these activities based on the comprehension of these processes. As the electrodes merely approach the membrane rather than penetrate it, this method would cause minimized disruptive effect on cell membrane and allows for a long-term recording continuing for weeks and months [29, 30]. Moreover, when microelectrode arrays are used to study a neuron network containing hundreds of individual neurons, multiple electrodes can provide adequate information, helping us to understand the connection pattern of neuronal circuits and their physiological function. To date, the number of electrodes in an individual microelectrode array in vitro is more than 10,000, mainly owing to the efforts to increase the density and the number of electrodes on a microelectrode array platform; and this enables the simultaneous stimulation and recording of a large group of neurons [14]. All these superior features make microelectrode array a robust electrophysiological method for investigations of a large population of neurons. However, compared to intracellular recording techniques, microelectrode arrays are always criticized for having several limitations. First, due to the extracellular recording mode, the microelectrode arrays have comparatively poor recording and stimulation quality. Extracellular recording minimizes the damage to recorded cells but also limits the efficiency of stimulating the cells [28]. To improve the signal quality and local stimulation strength, microelectrode array continues to increase the density and the number of microelectrodes integrated in a single microelectrode array, and just recently the high-density microelectrode array has successfully stimulated and recorded from single neuron [31]. Second, the electrodes stay in the extracellular environment and have no opportunity to record the subthreshold intracellular potential, which plays a vital role in signaling between neurons, such as excitatory- and inhibitory-postsynaptic potentials [32]. Third, the local field potential reflects the summated electrical activity from multiple cells, and it needs extra data analysis steps to distinguish signals responding to specific cells from others [14]. Therefore, precisely targeting specific cell regions using microelectrode arrays is indeed difficult. Finally, because the planar chip used in microelectrode arrays occupies a significant volume in vivo, the number of electrodes integrated in a single chip for in vivo studies is limited to nearly one hundred, much less than that in vitro [33].

## Current challenges of intracellular recording systems

### Parallelization

Further increasing the testing throughput of patch-clamp systems in vitro is necessary for many pharmacological and toxicological studies but this still faces many challenges. To explore the information processing and exchange in a specific region of the brain tissue, choosing a group of neurons statistically representing the local neuronal network and simultaneously monitoring and recording the changes in the membrane potential is a vital and practical approach. However, the gold standard method for intracellular recording, the patch-clamp method, is not suitable for multiplexing and only allows for less than 10 measurements simultaneously [28]. First, the volume and rigidity of the micropipette is the major limitation that prevents patch-clamp parallelization. The density of recording sites in patch-clamp systems using conventional micropipettes is limited by the large pipette devices. Second, many functions of the patch-clamp recording system, such as the suction application and pharmaceutical release, are derived from the microfluidic system connected to each pipette. However, the complexity of monitoring the microfluidic system in automated planar patch-clamp systems is an obstacle in developing highly parallelized systems and limits the number of pipette channels on a single recording platform [11]. The pipettes need to be cleaned after the recording or in each interval of the actions, but this process is highly complicated, and the simultaneous cleaning of a large number of pipettes is hard [20]. Currently, the commercially available automated patch-clamp systems in vitro have hundreds of recording sites and allow for a medium throughput electrophysiological experiment compared to fluorescence arrays and microelectrode arrays.

In terms of electrophysiological recording in vivo, there are more hurdles to parallelization than recording systems in vitro. The cells used in automated planar patch-clamp systems need to be isolated, suspended, and consistent in ion channel expression, which is highly different from the natural conditions in vivo [8]. The high throughput screening platform based on patch-clamps cannot be directly applied to cells in the tissue, has no selectivity in recording digested cells, and does not apply to neuronal network-level measurement. As for intracellular recording in vivo, the size of recording devices is more strictly limited to avoid mechanical damage to cells, and limitations derived from the pipette size and the fluidic system also exist [20]. Recently, a flexible quartz nanopipette has been applied in the stable intracellular recordings in mice and, as the tiny analogs of glass micropipettes used in patch-clamp recordings, this nanoscale electrode exceptionally reduces the diffusional flux and increases the stability of recording [34]. However, this system is still limited in the number of neurons that can be spontaneously recorded, mainly derived from the difficulty in controlling and densely positioning. So far, precisely controlling large quantities of glass micropipettes to keep their dense contact with the measured neurons has been proved to be hard and impractical. Therefore, the throughput of electrophysiological recording *in vivo* using a patch-clamp system is extremely low.

### Invasiveness

One of the characteristics of intracellular electrophysiological approaches is direct contact with the cytoplasm. This contact is established by the penetration of electrodes or pipettes through the cell membrane at various levels and thus is expected to result in an intrinsic disruption to natural neuronal physiology. In this respect, the invasiveness of sharp microelectrodes is larger than that of patch-clamps; in addition, it has been reported that penetration with a sharp electrode damages the membrane and causes significant depolarization. In contrast, patch-clamps cause only a series of action potentials soon after penetration, had no significant effect on the resting potential, and slightly reduced the input resistance of the recorded cell [27]. These observations confirmed the reliability of patch-clamps in short-term recording. Nevertheless, the application of patch-clamps in measurements lasting for several days and hours is considered to be unsuccessful due to the decay of intracellular signals. The interdiffusion between the pipette internal solution and the cytoplasm rapidly changes the transmembrane electrochemical gradients and the key functional molecules, leading to the disruption of the natural biochemical processes necessary for normal electrical activity [15, 35]. Unexpected dialysis negatively affects the long-term reliability of patch-clamp recording.

In comparison, patch-clamps in vivo have even more limitations in terms of invasiveness than patch-clamps in vitro. The highly invasive injection of micropipettes in vivo also causes serious dialysis due to the intrinsic disruption of the natural intracellular environment, affecting the concentrations of ions and functional molecules. Additionally, rigid micropipettes need to be connected with other electronic devices during insertion into the nervous system with a high-volume requirement. Most of the electronics used in the in vivo patch-clamp systems are highly rigid, and a slight mechanical movement of the experimental animals could remove the resistance seal and cause unexpected and irreversible damage to the recorded tissue [36]. To date, patch-clamps in vivo have been applied in living and fixed small animals to avoid mechanical perturbation. Moreover, considering that the devices used in recording in vivo still need to be delivered to the target region of brain tissue through invasive surgery, the in vivo application of patch-clamps is highly invasive [20].

### Spatial resolution

The scale of the micropipette is also a limitation for using electrophysiology in characterizing the electrical activities of small neurons and subcellular structures. Studies so far have measured the excitation of large neuronal compartments, but the application in smaller cells or microcompartments is unusual [37]. Generally, the conventional patch-clamp technique uses optical microscopy, such as differential interference contrast microscopy, to guide the pipette to the target position and form the seal. However, though many of the subcellular compartments are still larger than the optical limit of microscopy, the range of specific cellular structures that allow for measurement is limited by the tip size of the pipette [38]. Additionally, the large electrode structure also leads to a reduced spatial resolution that affects the ability to target specific cell types and subcellular structures in vivo. Recently, recordings from small neuronal structures have been obtained using neurons with fluorescence-labeled membrane proteins but have not been confirmed as being suitable for more cell types [38]. In summary, direct electrical access to the intracellular environment using a patch-clamp system has been limited to large structures of electrically excitable cells, such as the cell body, the large synaptic terminal, and the thick axons; leaving knowledge gaps in the functions and properties of the ion channels of small cellular structures.

## New opportunities created by nanotechnology

Because of the obvious limitations of conventional patch-clamp recording, the requirement of a massively parallelized, noninvasive, easy to control and high-precision intracellular recording platform (that is suitable for in vivo studies) motivates attempts to combine the present electrophysiological methods with nanotechnology. To date, nanotechnology has facilitated significant innovations in the areas of developing novel nanoscale electrical recording devices, characterizing the physiological status of recorded cells, and studying various interfaces between recording devices and the cell membrane. These changes represent the new progress of electrophysiology towards reliable, flexible, and efficient electrical recording from electrogenic cells.

### Design and production of electrical devices on the nanoscale

One of the most obvious advantages of using nanoscale electrical devices in intracellular recording is that the small size of the probe can significantly reduce the structural differences between the biomaterials and electronics and keep the homeostasis. New developments in nanofabrication have completed the production of a massive number of designs within the 1–100 nm level, including several one-, two- and three-dimensional nanostructured materials made from silicon, carbon, or a variety of metals [1, 39–41]. Using etching combined with chemical vapor deposition can easily fabricate large amounts of nanowires and nanotubes [16]. Through the bottom-up synthesis of functional nanowires on a planar substrate, a vertical and highly parallelized microelectrode array, which enables intracellular recording by directly penetrating the tips of nanowires into the recorded cells or electroporation, can be formed rapidly [42]. Moreover, the wide application of optical and electron beam lithography largely increases the fabrication precision, making patterning at the cellular and subcellular length scales routine. A recent study reported a successful attempt to introduce several regions of different doping types in a single nanowire to produce a nanoscale transistor, establishing the basis for nanoscale integrated recording devices [13]. Finally, it is practical to functionalize the surface of nanoscale probes with artificial biomaterials such as lipid layers, which would help to reduce the invasiveness in intracellular recording [43]. Therefore, nanofabrication technology makes the design of nanoscale noninvasive recording devices more convenient and shows significant potential in developing new intracellular recording systems.

### Characterization method on the nanoscale

The imaging and characterization methods used in nanoscience may help to achieve intracellular recordings with a high spatial resolution. Recently, an automated system using super-resolution hopping probe ion conductance microscopy has been proposed to measure the membrane potential of small central synapses, which permits the automatic scanning of identified cellular structures and seal formation. In the contrast, recordings on the subcellular compartments are extremely limited using traditional methods and previous attempts are mostly based on comparatively large structures [18]. However, the automated system uses fluorescence dyes to label the recycling synaptic vesicles and confirm the position of small synapses with an inverted laser-scanning confocal microscope. The system has nanoscale pipettes and enables the evaluation of the distance to the cell membrane by measuring the changes in the pipette current under a constant voltage; meanwhile, the resolution of the evaluation depends on the diameter of the nanoscale pipette [38]. A group has created a plasmonic biosensor based on nanoscale pipettes, similar to those used in patch-clamp systems, via decorating the pipettes with Au nanoparticles to create a specific surface for surface-enhanced Raman scattering analysis. This device can be used to monitor the local metabolite concentrations close to the detection zone and has obtained several successful recordings near living mammalian cells in a previous study, which show the potential of being integrated with patch-clamps to explore more physiological mechanisms [41, 44]. In addition, high-resolution characterization methods are necessary in the design and production of new nanoscale structures and also important in studies of the interaction between nanoprobes and the cell membrane. For example, high-resolution examination of the nanotube-cell interface has been used to explain the enhancement of the amplitude and the prolongation of the recording duration in intracellular recording with nanotube electrodes and further facilitates studies on the relationship between the membrane curvature and the recovery duration after electroporation [2].

### Nanomaterial‐cell interface

For intracellular electrophysiological recording, specific engineering of the nanoscale interface between biomaterial and artificial devices is a potential direction for improving the recording quality. Compared to conventional pipette electrodes, nanoscale electrodes have many unique features enabling special interactions with the cells, including the engulfment, adhesion, and modulation of the ion channel expression [36]. By increasing the seal resistance between the patch-clamp pipette and the cell membrane, intracellular recordings can avoid perturbation from the membrane shunt after penetration and achieve an increased signal-to-noise ratio [45]. In this context, several attempts have been made to functionalize the surface of nanoscale probes to integrate them into the lipid bilayers of the recorded cells. This approach is considered promising because it reduces the invasiveness, locally modulates the property of the cell membrane, and stabilizes the seal in long-term recording [15]. The examination of the narrow interface of electrodes with the cells resulted in a positive engulfment behavior of recorded cells when contacted with nanopillar-shaped electrodes. This engulfment mechanism reduces the distance between the extracellular electrode and the cell surface, leading to an enhanced seal [1]. Several groups have proposed many special three-dimensional designs of nanoelectrodes to utilize this mechanism in extracellular recording, aiming to achieve output signals similar to intracellular recordings [17, 46]. Moreover, the capability of a novel nanoscale film made of hydrotalcite-like compounds to adjust the expression of potassium channels and aquaporins in astrocytes has been reported recently. This nanoscale biocompatible material promotes the differentiation of astrocytes in vitro, probably underlying the development of new in vitro models to study astrocytes [47]. In summary, studies of material-cell interfaces reveal several practical interaction mechanisms for the improved performance and extended application of recording systems.

## New trends in intracellular recording systems using nanotechnology

**Fig. 2:**
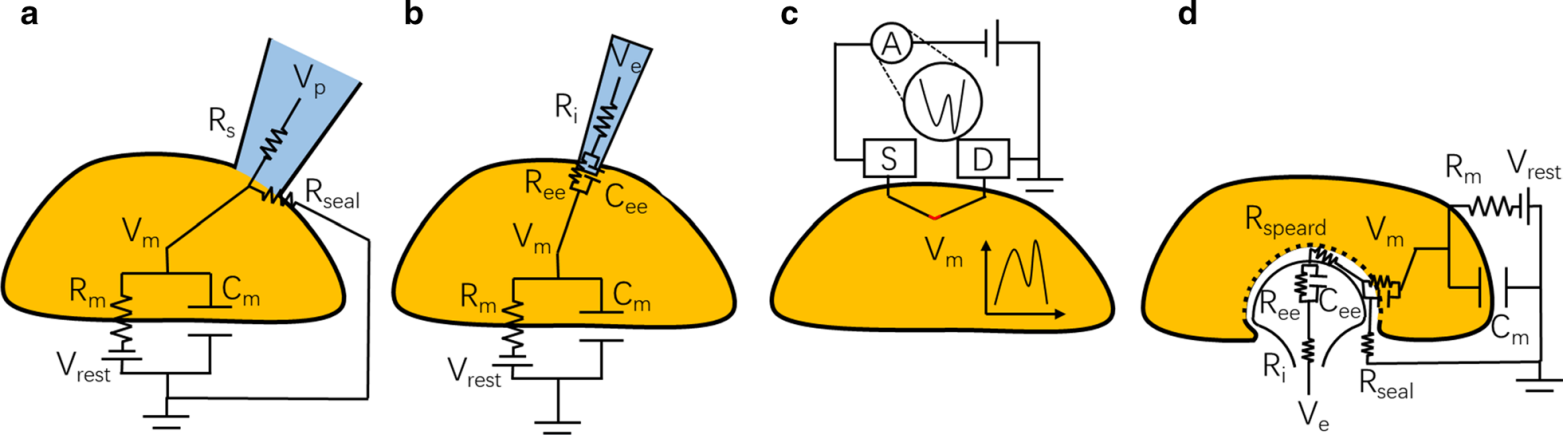
Equivalent circuits. (adapted from ref.[57]) of the intracellular recording from **a** Patch-clamp pipette; Here, ${V}_{m}$, ${C}_{m}$ and ${R}_{m}$ denote the membrane potential, capacitance and resistance, respectively; ${V}_{rest}$represents the rest potential; ${R}_{seal}$represents the seal resistance; ${R}_{s}$represents the resistance in series; ${V}_{p}$represents the signal recorded by the pipette. **b** Nanoelectrodes based on direct electrical contact with the cytoplasm; Here, ${R}_{ee}$ and ${C}_{ee}$ denote the resistance and capacitance of the interface between the microelectrode and the electrolyte solution, respectively; ${R}_{i}$represents the electrode impedance and ${V}_{e}$ reflects the signal recorded by the electrode. **c** Nanoscale field-effect transistors; Here, the symbol “S” and “D” represent the source and drain components, respectively. **d** Micro-mushroom-shaped nanoelectrodes. Here, ${R}_{spread}$ represents the resistance encountered by the current spreading from the microelectrode surface to the electrolyte solution

Due to the development of technologies to fabricate and assemble electronics on nanoscale, diverse novel electrophysiological recording platforms were proposed. The intracellular recording systems based on these new tools had excellent performances in measurements of membrane voltage and brought about several innovative changes to related studies, probably representing the developing directions of the future recording approaches. Several common configurations of patch-clamp recordings and recordings from nanoscale electronics have been presented in Fig. 2.

### Nanoelectrodes relying on direct electrical contact with the cytoplasm

**Fig. 3:**
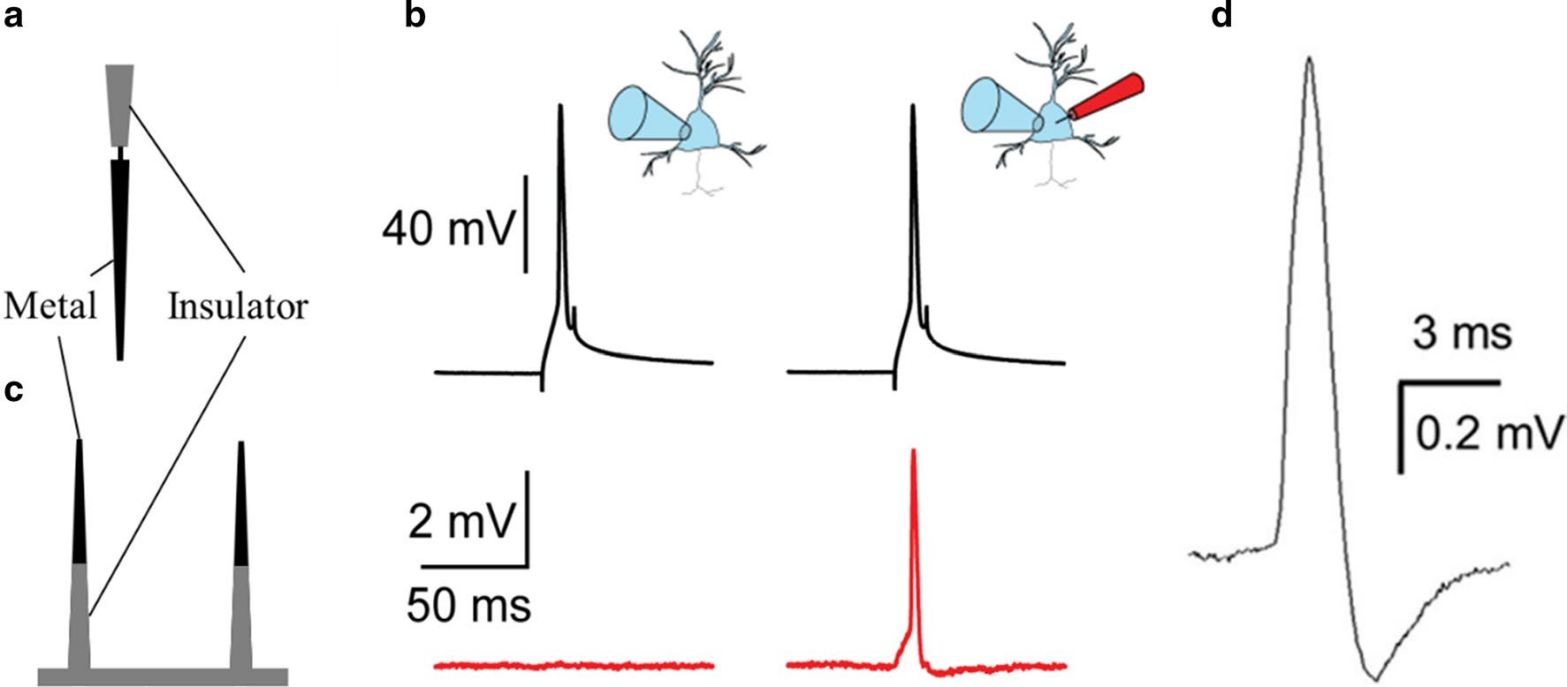
Nanoelectrodes based on direct electrical contact with the cytoplasm and their recording capabilities. **a** Schematic diagram of the free-standing solid-conductor intracellular nanoelectrode, adapted from ref. [1]. **b** Spontaneous action potential recordings by a whole-cell patch-clamp micropipette and a nanoelectrode, reproduced from ref. [1] The action potentials recorded by the nanoelectrode (red arrow) is similar to that recorded by the whole-cell patch-clamp. **c** Schematic diagram of the vertical nanowire electrode array, adapted from ref. [12]. **d** An intracellular-like spike recorded by nanoelectrode array platform after optoporation, reproduced from ref.[49]

The sharp microelectrode is a typical traditional recording device in electrophysiology but the direct insertion of electrodes would cause serious damage to cells. Derived from the requirement of a less invasive recording device, the solid electrodes consist of nanowires or nanopillars have been developed. Previous studies have reported applications of several nanomaterials in the fabrication of electrodes designed to record transmembrane potential, including metals, silicon, and carbon [1, 30, 39, 40]. The design of metallic nanoelectrodes is similar to that of conventional sharp electrodes that exchange ions with the cytoplasm through a direct penetration (Fig. 2b). The ion beam is used to mill the tungsten microelectrodes and construct solid metallic intracellular nanoelectrodes with diameters ranging from 100 to 400 nm and lengths of several micrometers (Fig. 3a). The recordings with intracellular metallic nanoelectrodes report lower amplitude data compared to conventional whole-cell patches while the nanoelectrode and the patch-clamp measure 2.62 mV and 109 mV for the same feature, respectively (Fig. 3b) [1]. However, the shape of the transmembrane potential recorded by metallic nanoelectrodes is similar to that from whole-cell patch-clamp recording (Fig. 3b). The invasiveness of novel nanoelectrodes is significantly lower than that of conventional microelectrodes, but over 90 % of penetration behaviors still affect the cellular physiology, represented by the reduced input resistance and membrane potential. The access to transmembrane potential achieved in metallic nanoelectrode recording disappeared over periods of seconds to minutes, significantly reducing the potential for application in long-term recording [1]. Silicon-based nanoscale electronics have several special features that attract the attention of electrophysiologists, such as unique mechanical properties, adjustable electronic and optical properties, and high biocompatibility [36]. The fabrication of silicon-based electrical devices is well studied owing to the development of electronics and integrated circuits [10]. In early attempts, large-scale and high-density patterned silicon probes have been used when recording local field potentials with high temporal resolution while the novel nanoscale probes further enable the recording of individual neurons with a high spatial resolution. Researchers constructed silicon nanowire electrodes in a top-down manner on a planar substrate to establish a vertical nanowire electrode array (Fig. 3c). One single small electrode array can contain 9 or 16 silicon nanowire cores with silicon dioxide shells, and the whole area is smaller than the typical size of the cell body of a neuron, permitting single-cell coupling [12]. One of the advantages of this recording platform is that the electrode array is scalable, and it is easy to parallelize multiple identical devices to record hundreds of neurons simultaneously. This platform relies on electroporation to obtain intracellular signals, which may damage the recorded cells but truly increase the signal amplitude (Fig. 3d). This electrode array platform can easily record presynaptic and postsynaptic neurons without the identification of each cell under optical microscopy. Carbon-based electrical devices are a promising direction, but limited conclusions have been made in this context. It has been reported that different electrode materials significantly affect the signal-to-noise ratio, and the graphene microelectrode arrays fabricated by chemical vapor deposition show an obviously increased signal quality compared to the metallic materials [40].

The ways nanoelectrodes are used to establish stable intracellular access include mechanical penetration, spontaneous poration and internalization, electroporation, and optoporation. Mechanical penetration is the most direct way to achieve intracellular access, but it is considered to be invasive and is rarely applied in actual measurements. The sharp tip of the nanoelectrode may spontaneously enter the recorded cell without any disruption to cellular properties, but this spontaneous insertion is extremely rare in practice. Therefore, several strategies for surface functionalization to facilitate the internalization of nanoelectrode recording sites have been proposed, and the most common approach is phospholipid modification, which could significantly reduce the disruption through lipid layer fusion [1, 15]. Moreover, electroporation is one of the most commonly used methods to improve the intracellular recording signal of nanoelectrodes. By applying localized electric pulses, the impedance between the cytoplasm and electrode can be lowered by several orders of magnitude, thus improving the recorded signals. The effect of electroporation lasts for several minutes, and the recorded signal amplitude decays with the recovery of the cell membrane [48]. Several groups demonstrate that electroporation needs to inject currents into the recorded cell and potentially perturb the spontaneous electrical cell activities. Therefore, researchers developed optoporation, which applies short laser pulses at the tip of the nanopillars to create transient nanopores into the cell membrane and access the intracellular environment [49].

Compared with conventional microelectrode arrays, nanoelectrode arrays have a higher spatial resolution and can be packed more densely. Due to the small electrode tip size at the subcellular level, cells may positively attach and engulf the protruding tip and establish a powerful interface with nanopillar electrodes. This strong interaction between the cell membrane and electrode promotes the spontaneous internalization of the nanoelectrode and enhances the resistance seal, achieving increased signal quality [48]. Based on this tight junction with the recorded cell, the nanoelectrode can establish a one-to-one correspondence between the cell and electrode, thus addressing the issue of unclear signal source attribution in the practices of local field potential recording [32]. Additionally, the production of nanoelectrode arrays can be easily integrated with CMOS chips to facilitate large-scale parallelized recording [11]. This strategy prevents the direct connection between the sensing electrode and electronics and reduces the effect of electrode impedance on the recording quality. To date, the combination of nanoelectrode and CMOS technology has extended the number of recording sites to 1024 and achieved simultaneous recording from hundreds of neurons [10].

One of the recent advances in prolonging the recording duration of intracellular signals in terms of vertical nanoelectrode arrays was made by Lin et al. [2] who proposed a novel design using nanotubes. The nanotube electrode could also be engulfed by the cell membrane, and the hollow core of this structure simultaneously allowed for the bottom membrane to protrude into the nanotube. This interaction enhanced the cell-electrode interface and significantly increased the quality of the recorded intracellular signal. In addition to the increased signal quality, the section of the bottom membrane inside the nanotube had a highly positive curvature, which could affect the recovery of membrane electroporation and prolong the intracellular access duration by 1–2 orders of magnitude [2].

### Nanoscale field‐effect transistor

**Fig. 4:**
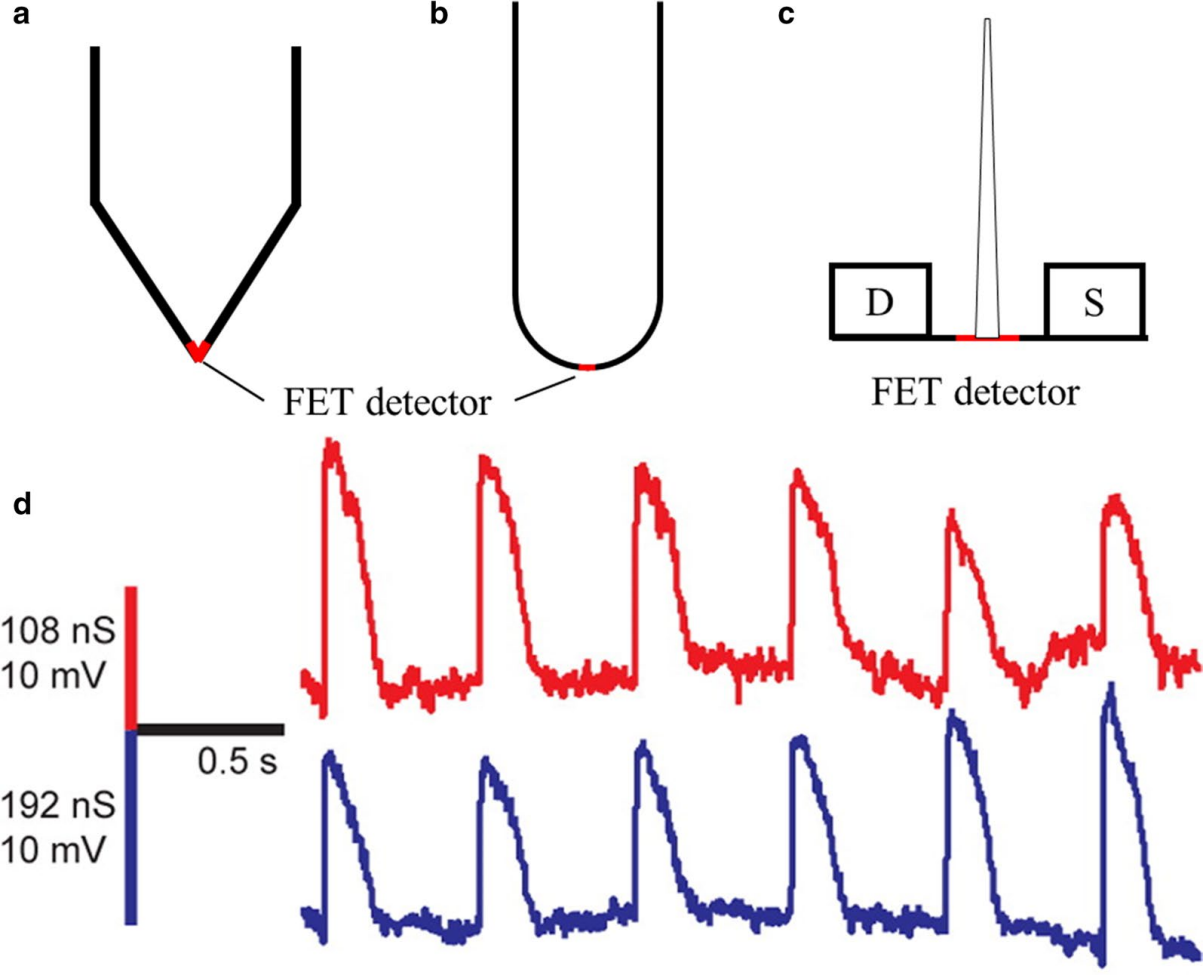
Nanoscale field-effect transistor electrodes. **a** Schematic diagram of the free-standing V-shaped nanotransistor electrodes, adapted from ref. [13]. **b** Schematic diagram of the U-shaped nanotransistor electrode, adapted from ref. [51]. **c** Branched intracellular nanotube field-effect transistor, reproduced from ref. [55]. **d** The action potential recordings from a free-standing nanotransistor probe in contact with a cardiomyocyte, being represented as an intracellular recording, reproduced from ref. [50]

In the practice of nanowire or nanopillar electrodes, an intrinsic limitation is the compromise between the increasing recording quality and reducing invasiveness because most of these electrodes need to directly exchange ions to measure the membrane potential. Although continuously reducing the diameter of the recording electrode is significantly helpful for minimizing the invasiveness, the corresponding increased electrode impedance would adversely affect the signal quality in such an electrical circuit [15, 50]. To solve this problem, several groups have begun to use nanoscale FETs to achieve high-precision intracellular recording. Tian et al.  [13] reported a novel fabrication strategy to adjust the semiconductor composition during synthesis and incorporate several different functional regions in a single nanowire. By changing the conditions during growth, kinks could be introduced into silicon nanowires, and the arms of the kinked nanowires could be connected to the source and drain components. The tip of this kinked nanowire is doped to create a light n-type doping region as the detector zone of the nanoscale FET (Fig. 4a). The nano-FET probes obtain the signal of the transmembrane potential by analyzing the current between the source and drain instead of keeping the direct ion exchange with the cytoplasm, thus minimizing the probe size without considering the impedance (Fig. 2c). This nanoelectronic device has been used to measure the membrane voltage of embryonic chicken cardiomyocytes and reported similar amplitudes, signs, and durations of the membrane potential as those from whole-cell patch-clamps [13].

By improving the fabrication framework used in nanowire transistor production, nanowires with different shapes and recording properties are obtained, providing multiple probe designs for several complicated applications in neuroscience. In addition to the V-shaped original design of nanowire transistors, several new types of functional nanotransistors have been reported, including U-shaped, W-shaped, and some integrated structures. Zhao et al. first presented the fabrication of U-shaped nanowires using a U-shaped trench to define the shape of the final product (Fig. 4b) [42]. During fabrication, straight nanowires were added to a specific substrate with lithographically patterned trenches and covered completely by high viscosity mineral oil to provide enough shear force. When the substrate was linearly translated, the straight nanowires were tightly anchored in the trenches and obtained the same specific shape as that defined by the trenches. Therefore, this approach achieves high-precision control of the nanowire geometry and allows for large-scale assembly. Based on this advanced fabrication strategy, U-shaped nanowires were rapidly converted into nano-FETs by introducing a metal contact at the tip of the nanowire and producing a transistor sensing region (Fig. 4b) [51]. The application of U-shaped nanowires in dorsal root ganglion (DRG) neuron recordings showed a representative intracellular-like action potential signal (Fig. 4d). There was a significant relationship between the radii of the curvature and the recorded maximum amplitudes, which reflects the capability of nanoscale tips to induce endocytosis. This U-shaped probe has showed its potential in parallelization because the arms of this probe were parallel and required less volume than the original V-shaped design [52]. The fabrication strategy of W-shaped nanowire probes was proposed based on the gold nanoparticle catalyzed vapor-liquid-solid growth method, and the W-shaped nanowires would have two FET sensing elements, allowing for simultaneous recording [45]. The W-shaped nanowires provide an opportunity for the use of independent free-standing nanowire devices *in vivo* to improve the capability of multiplexing by delivering multiple devices with an individual probe. However, the signal amplitudes of the two recording sites were limited by the geometrical structure of the W-shaped probe because making two FET detector regions simultaneously reach the cytoplasm requires the probe to move deeper from the surface [50].

As described above, nanowire transistors were not only applied in large-scale integrated arrays, but they were also used in the design of small, independent, and free-standing probes. Compared to designs based on planar substrates, these independent nanoscale probes could target specific cells and subcellular structures via high-precision manipulation similar to that used in conventional patch-clamps. Moreover, several intrinsic limitations of kinked nanowire transistor arrays were avoided, including the high spatial limitation in the arrangement caused by the kinked configuration of the nanowires and the determined sensor positions during fabrication with reconfiguration difficulties. To date, free-standing nanoprobes using U-shaped nanowires have obtained an extremely small detector size *in vivo*, achieving a tight membrane seal and prolonging the stable recording duration [33].

In addition, several groups have demonstrated that applying nanotubes in field-effect transistor production or combining the current comprehension of the two nanodevices to create new designs is worth studying [53]. Fahad et al. highlighted the limited area efficiency and increased fabrication difficulty of conventional nanowire transistors and proposed a FET with a nanotube structure. The application of this nanotube transistor could achieve desirable electrical characteristics and significantly improve the performance of individual FET detectors in a large-scale array [54]. Regarding efficiency, this design could be an ideal substitute for nanowire FETs. In parallel with the development of the nanotube transistor, efforts to combine the nanowire FET and nanotubes also resulted in the proposed novel design of a nanoscale recording device, the branched intracellular nanotube field-effect transistor (Fig. 4c). This method integrated the bottom of the insulating SiO_2_ nanotube with the detector region of the original nanowire transistor. The tip of the nanotube penetrated the cell membrane and established a contact between the cytoplasm and the FET detector region through the hollow center of the nanotube, thus allowing for additional reducing the size of the probe inserted into cells [16]. In this case, the attempts to further miniaturize the probe faced several important limitations in maintaining a high sensitivity and signal-to-noise ratio because the inner diameter of the nanotube defined the effective detection area of the FET, and the reduction of nanotube diameter would lead to reduced sensitivity and increased solution resistance in the nanotube. To overcome the above limitations for miniaturization, researchers created special nanotube probes containing a thin top tip and a thick bottom base by selectively etching the top section of the SiO_2_ nanotube. Therefore, the production of intracellular recording devices using nanotubes with tip sizes at the sub-10 nm level is possible. The application of this recording device in the action potential measurement of cardiomyocytes has reported a full amplitude intracellular recording, and the continuous duration could exceed one hour, showing significantly high signal quality and low invasiveness [55].

### Recording electrodes relying on dense attachment

**Fig. 5:**
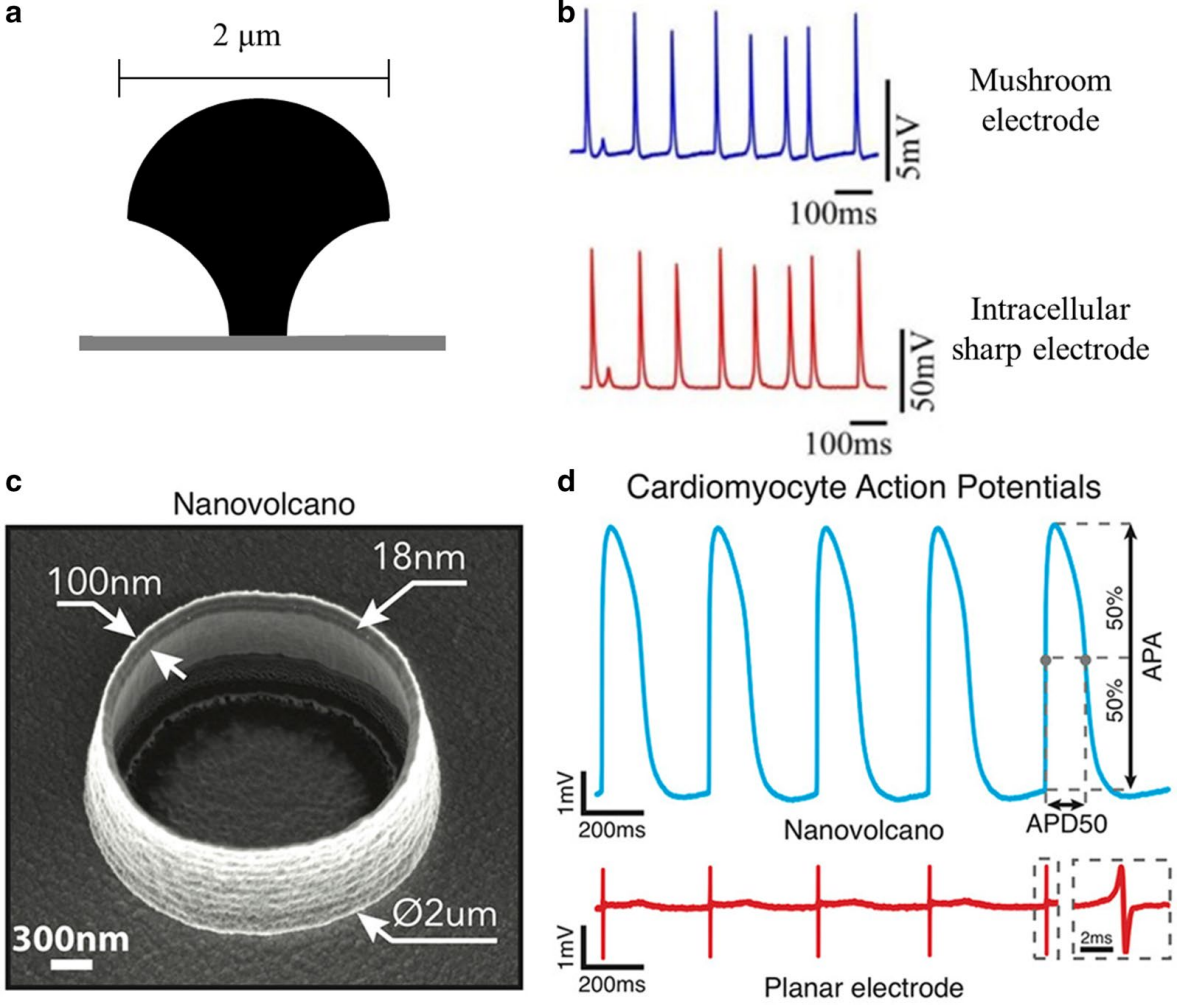
Intracellular-like extracellular recording electrodes. **a** Schematic diagram of the micro-mushroom-shaped electrodes, adapted from ref. [46]. **b** The simultaneous recordings of action potentials from a single Aplysia neuron by the micro-mushroom-shaped electrode and an intracellular sharp electrode, reproduced from ref.[32] The consequence showed identical firing patterns. **c** Nanovolcano-shaped electrode, reproduced from ref. [17]. **d** The action potential signals recorded by a nano-volcano electrode from a rat cardiomyocyte with a typical intracellular shape, reproduced from ref. [17]

The microelectrode array has minimized the invasiveness to cells because the device does not destroy the integrity of the cell membrane, but this characteristic also prevents the microelectrode array from obtaining intracellular signals. Recently, several advances in analyzing the cell-electronic interface have been developed as the basis of a new design strategy to develop novel extracellular recording electrodes that can obtain stable intracellular signals (Fig. 2d). Notably, extracellular nanopillar electrodes can be engulfed by the cell membrane, thus enhancing the resistance seal. In this context, a special electrode design based on neuronal topology probably offers the opportunity to record transmembrane potential using extracellular electrodes by minimizing the seal conductance [56]. The structure of the novel electrode was similar to a micro-mushroom and designed to promote the interaction between cells and electrodes, allowing for embracement by the neuronal cell body (Fig. 5a). Several groups have proven the capability of this new electrophysiological tool to achieve high-quality extracellular recordings [46]. In order to transform the recorded extracellular signal to transmembrane potential, the micro-mushroom electrode induced the embracement of the recorded cell to stretch the membrane and increased the membrane conductance. The application of micro-mushroom electrodes in the recordings of Aplysia neurons reported an attenuated action potential and postsynaptic potential with classical intracellular recording characteristics (Fig. 5b) [32]. The equivalent circuit simulation of the signal recordings by the extracellular mushroom microelectrode revealed that simultaneously increasing the seal resistance and the transmembrane conductance of the section of membrane coupled to the electrode could switch the shape of the recorded potential to the transmembrane potential [57]. In summary, although the amplitudes recorded by mushroom-shaped microelectrodes were low compared to the full amplitude recordings using conventional patch-clamps, this approach provided adequate information about the types, statuses and properties of the ion channels on the membrane, enhancing our comprehension of several subthreshold electrophysiological activities in a noninvasive manner.

In addition to mushroom-shaped microelectrodes, several other designs of nanopatterned extracellular recording electrodes have been proposed or await optimization. In a previous attempt to record transmembrane potential with the nanotube electrode combined with electroporation, it was reported that the hollow center of the nanotube led to the positive curvature of the membrane inside the nanotube, which accounted for the decreased gap distance between the membrane and electrode [2]. The section of the membrane with a positive curvature had a higher membrane tension than other sections and helped to obtain intracellular signals. A nanopatterned volcano-shaped microelectrode utilized this mechanism to optimize the membrane-electrode interface and increase the opportunity to obtain stable intracellular access (Fig. 5c) [17]. Because the electrode was designed to contact the cell and induce a high curvature region instead of penetrating the cell, the size of the electrode can be expanded to reduce the impedance and be integrated with CMOS chips to optimize parallelization. The recordings of intracellular action potential from rat ventricular cardiomyocytes reported a typical transmembrane potential with an attenuated amplitude (~ 20 mV) and a prolonged recording duration, as demonstrated by a previous study using a nanotube electrode (Fig. 5d) [17].

### Current challenges of nanodevice‐based intracellular recording

**Fig. 6:**
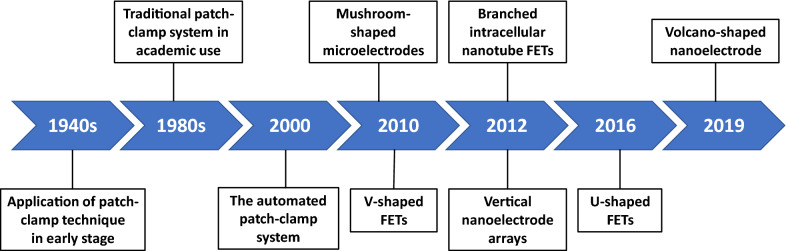
A timeline showing the development of devices and platforms used in intracellular recordings. The time nodes listed in the timeline represent the first time the corresponding devices or platforms are reported. Data sources can be found in Table 1

As described above, new electrophysiological intracellular recording methods based on nanotechnology have achieved obvious advances over conventional patch-clamp techniques regarding invasiveness, parallelization, and spatial resolution. The development of intracellular recording methods is showed in Fig. 6 and a comparison of the performance of the common intracellular recording systems is presented in Table 1. However, these developing approaches, including nanopillar arrays, nanoscale FETs, and extracellular noninvasive electrodes, are still limited in several aspects. In terms of nanowire and nanopillar electrodes that directly exchange ions with the cytoplasm, first, the impedance of the electrodes could be the most important limitation to the size of the electrode, which reduces the signal-to-noise ratio as the cost of less invasiveness (Fig. 2b). In the contrast, the recording systems using electrolyte-filled electrodes and FET electrodes hardly cared about this limitation. Second, although nanoelectrode arrays could significantly increase the testing throughput and achieve high efficiency in analyzing neuronal network activity in vitro, the application of these arrays in vivo was extremely limited, owing to the volume requirement and difficulty in targeting specific cell types [33, 50]. In addition, the relative position of the recording electrodes with a nanoelectrode array was fixed during manufacturing, suggesting that changing the position of the recording device to suit the distribution of neurons during the experiment is impossible. Moreover, nanoelectrodes that exchange ions with the cytoplasm would inevitably lead to current leakage and a membrane shunt. By increasing the seal resistance, the current leakage can be minimized, but the reduction of the membrane shunt requires the application of a high impedance electrode, thus affecting the recorded signal, especially for high input resistance cells. Finally, as reported in previous studies, the seal resistance between vertically arranged nanoelectrodes and HEK293 cells (100–500 mΩ) was lower than the standard values using a conventional patch-clamp system (almost not less than 2 gΩ), which requires improvement [12].

**Table 1:** The performance of the common intracellular recording systems

Name	Electrode size	Cell type	Seal resistance	Recorded maximum amplitude for AP	Parallelization	Pathways for intracellular access	References
Patch-clamp	~ 4 μm	Neurons (from *Xenopus* tadpoles)	> 1 gΩ	80–100 mV	< 10	Mechanical penetration	[27]
Vertical nanowire electrode arrays	150 nm	Neurons (Rat cortical neurons)	100–500 mΩ	~ 5 mV	Scalable	Electroporation	[12]
Nanopillar metallic electrode	100–400 nm	Neurons (Brain slices)	N/A	~ 2.5 mV	None	Mechanical penetration	[1]
V-shaped FETs	~ 20 nm	Cardiomyocytes	N/A	80 mV	Scalable	Mechanical penetration	[13]
U-shaped FETs	50 nm	Neurons (Dorsal root ganglia)	N/A	5–20 mV	Scalable	Mechanical penetration	[51]
Branched intracellular nanotube FETs	~ 8 nm	Cardiomyocytes (HL-1 cell)	N/A	75–100 mV	Scalable	Mechanical penetration	[55]
Mushroom-shaped microelectrode	~ 2 μm	Neurons (*Aplysia*)	> 100 mΩ	2–30 mV	Limited by electrode size	Endocytosis	[32]
Volcano-shaped nanoelectrode	~ 2 μm	Cardiomyocytes (Rat cardiomyocytes)	> 100 mΩ	1.5–20 mV	Limited by electrode size	Endocytosis	[17]

The function of nanoscale FETs did not rely on ion exchange with the intracellular solution, thus allowing for a smaller electrode design and less invasive injection. Though this electrophysiological tool has exhibited excellent performance in intracellular recording, it still has several limitations that prevent it from being widely applied. First, the nano-FETs could not deliver molecular reagents to the recorded cells during an experiment and require extra pipettes integrated into the system to support drug applications, which is an obvious limitation to their use in drug discovery and screening [33]. Second, FETs have no ion exchange with the cytoplasm, suggesting that activating stimulation through direct injection currents is impractical (Fig. 2c) [14]. Coupling the nanowire FET detector with an activating electrode may be a solution, but this also increases the complexity of the design. Third, the fabrication of nano-FETs may be complicated and costly. The fabrication strategy of silicon nanowires plays an important role in the production of FETs, but it always applies gold nanoparticle catalysts to control nanowire growth, which significantly increases the expected cost of the system [53].

Regarding the extracellular recording electrode, the attenuated signal amplitude, which may be ignored by the recording device as a result of decayed intracellular signal, could limit studies of the subthreshold potential. In addition, although the present mushroom-shaped microelectrodes have been used to record large Aplysia neurons, it has not been confirmed that this electrode can also record the transmembrane potential of other neurons, especially small neurons. This is because the size of the microelectrode is so large that only the cell body can engulf the electrode and enhance the seal while small neurons and subcellular structures may not be suitable for the application of this electrode (Fig. 2d) [17]. Moreover, the extracellular recording electrode relies on the increased membrane conductance to achieve intracellular access, thus reducing the efficiency in recording high input resistance cells [32].

## Concluding remarks and perspectives

**Fig. 7:**
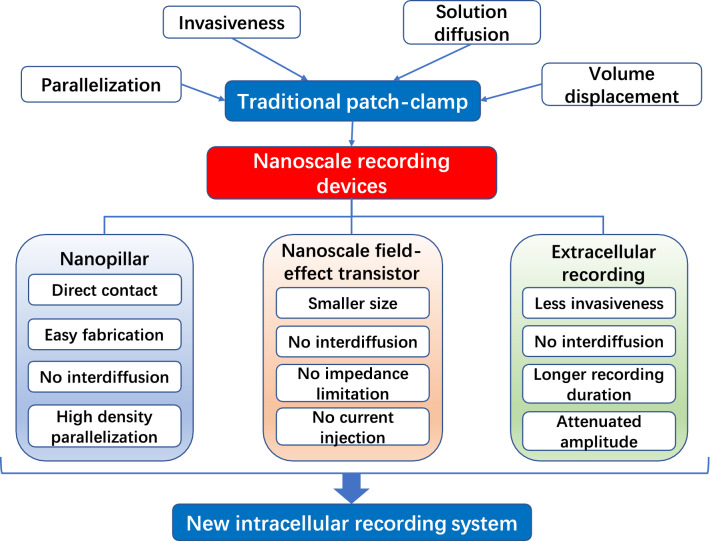
Nanoscale electronics for intracellular recording. Various nanoscale devices have been developed to record transmembrane potential, including nanoelectrodes made of nanopillars or nanowires that rely on the direct contact to the cytoplasm to record membrane potential, nanoscale field-effect transistors that connect the cytoplasm with the detector zone of the electrodes, and extracellular recording electrodes that reported intracellular-like signals

The conventional patch-clamp technique has long been the gold standard for intracellular electrophysiological recording methods. Despite the high-precision and high-temporal resolution information provided by the patch-clamp system, this traditional tool was critiqued for possessing several limitations in practice, and nanotechnology-based strategies have been proposed to solve these issues and develop a powerful, non-invasive and adaptable intracellular recording system (Fig. 7). By using high-resolution characterization techniques to study the interface reaction between the cell and recording device, we can provide new insights into how transmembrane potential can be recorded and guide the design and production of novel nanoscale electrodes.

Over the past few decades, several new intracellular recording techniques based on the development of nanofabrication and nanopatterning have been proposed, and these techniques have achieved success compared to conventional techniques at various levels. These approaches can be classified into three major groups according to the principles behind their recordings, which are the nanoelectrodes exchanging ions with the cytoplasm, the nanoscale FETs with a specific detection area, and the extracellular recording electrodes that output attenuated intracellular-like signals. Nanoelectrodes obtain intracellular access by directly penetrating the cell or electroporation and optoporation. The reduced disruption of the cell physiology by nanoelectrodes is attributed to the small electrode tip size, but the design of smaller electrodes faces compromises between the signal quality and reduced electrode size. The performance of nanowire FETs is not limited by the impedance, thus achieving significant advantages in long-term recording and parallelization. However, the fabrication of this device is complex and costly, and the reagent delivery and stimulation activation functions are limited. The extracellular recording electrode maintains the completeness of the cell membrane and simultaneously can obtain attenuated intracellular signals by forming a tight seal with the cell. This is an interesting and promising strategy, especially for those cases in which the cellular physiological status must be protected, such as recordings that last for days and weeks. However, the signals obtained by this technique were weakened, and the technique is therefore not suitable for studies during which the amplitude frequently changes because of the reduced precision. The design of 3D structures of the nanoelectrode arrays attracts much attention, attempting to make new recording devices ultra-flexible and biofriendly [58]. These devices are expected to be easy to implant and be friendly to the nervous system, avoiding the potential immune reaction to the utmost extent [59]. Based on the discussion above, all these approaches have achieved various advances over the conventional patch-clamp technique, representing new developments in recording methods for transmembrane potential measurement.

Regarding the application of intracellular recording methods in vitro, an outstanding system needs to have multiple positive features. It is expected that such a system would be suitable for most cells, either large or small, and the smaller subcellular compartments. This system should be easy to manipulate and cause little perturbation of the recorded cells. The overall recording system should permit long-term recording and include massive electrodes to record signals from multiple isolated cells or a cell network. According to the criteria listed above, nanoelectrode arrays using nano-FETs would be the best choice for developing such an in vitro recording system. Of course, several technical issues exist, including the density of the nanowire FETs on the substrate and the intracellular access achieved by injection. The kinked configuration of the original design of nanowire FETs is a limitation of the distance between adjacent nanowire detectors [16]. However, the fabrication strategy of U-shaped nanowire FETs allows researchers to define the ratio of the curvature of the electrode tip and the distance between the two arms of the FET probe, thus addressing the issue of the spatial distribution [51]. This alteration makes the arrangement of FET probes on the recording chips more flexible and gives more positions for the supporting devices, such as the stimulating electrode. In terms of invasive injection, several functionalization methods to promote the natural internalization of nanoscale electrodes by cells have been proposed. By covering the surface of nanoscale electrodes with phospholipid bilayers, the disruption of injection to the cell membrane potential was minimized [13]. The combination of these novel technologies has been an exciting achievement in intracellular electrophysiology, forming the basis of a revolutionary, non-invasive, high-throughput in vitro intracellular recording technique.

However, the in vivo recording system faces far more issues than the in vitro system. One of the crucial problems of in vivo recordings is volume displacement, which prevents the massively parallelized nanoelectrode arrays used in vitro from being applied in in vivo studies. Since nanoelectrode arrays have difficulty targeting specific cell regions in vivo, the development of free-standing nanoelectrode probes may be a more attractive choice. Additionally, the electronics used in vivo need to be flexible to avoid potentially damaging to soft brain tissue in the recording. However, in most cases, the surgery needed to place the overall recording terminal into specific regions of the brain damages the experimental animal. The major challenges of the in vivo recording system include three parts: placing recording devices into the target region of the brain, keeping the electronics in the brain tissue working stably, and developing a method to end the recording without extra damage. Recently, the exploration of in vivo intracellular recording systems using biocompatible and flexible nanomaterials has achieved many valuable results. Flexible nanowire FET detectors are mechanically compliant, capable of decreasing the damage to the brain tissue derived from the mechanical motion of the experimental animal and ensuring the stability of the recordings [15]. This characteristic is extremely important for long-term in vivo measurements. Besides, the ultra-flexible structures may permit direct injection by a syringe, thus maximally reducing the invasiveness of surgery [36]. The application of a series of nanoagents that can precisely target the specific regions in vivo provides a further strategy that these ingenious nanoscale designs could be used to enter human body and reach the nervous system without additional damage [60]. A recent study demonstrated that the structural, mechanical, and topological differences between natural brain tissue and artificial recording devices were the fundamental issue underlying the difficulty in long-term in vivo recordings [29]. Due to the topological distinction, conventional probes for intracellular recordings in brain tissue would displace and exclude endogenous neuronal tissues and, at the neuronal network level, disrupt the interaction between neurons and glia, leading to a chronic adverse immune response. Therefore, despite the minimal invasiveness of recording to an individual cell, some adjustments to the overall recording system possibly consisting of multiple individual electrodes are necessary. A macroporous interconnected structure allowing for interactions and direct contacts between neurons and other supporting cells without altering their endogenous distribution, which is prohibited by the plat substrate, has been proposed. This topology-based recording system may have an unprecedented ability to record over a long timespan [61]. Finally, safely removing the implanted electrodes would be a perfect end to successful in vivo recording, and bioresorbable materials represent an attractive solution. Silicon-based electronics can be spontaneously degraded through hydrolysis into silicic acid (Si(OH)_4_), but this process needs precise control to ensure the complete function of the electrode before degradation [29]. In summary, there are still many technical obstacles to a complete in vivo recording system, but considerable progress has been made, and the system shows great promise for intracellular recording technologies in vivo.

## Data Availability

All data related to the manuscript are available in the manuscript in the form of tables and figures.
